# Disentangling False Memories: Gray Matter Correlates of Memory Sensitivity and Decision Bias

**DOI:** 10.3390/neurosci6030068

**Published:** 2025-07-23

**Authors:** Ryder Anthony Pavela, Chloe Haldeman, Jennifer Legault-Wittmeyer

**Affiliations:** Department of Psychology, Elizabethtown College, Elizabethtown, PA 17022, USA; pavelar@etown.edu (R.A.P.); haldemanc@etown.edu (C.H.)

**Keywords:** false memory, gray matter, structural magnetic resonance imaging, parietal lobe, occipital lobe

## Abstract

Human memory is inherently susceptible to errors, including the formation of false memories—instances where individuals mistakenly recall information they were never exposed to. While prior research has largely focused on neural activity associated with false memory, the structural brain correlates of this phenomenon remain relatively unexplored. This study bridges that gap by investigating gray matter structure as it relates to individual differences in false memory performance. Using publicly available magnetic resonance imaging datasets, we analyzed cortical thickness (CT) in neural regions implicated in memory processes. To assess false memory, we applied signal detection theory, which provides a robust framework for differentiating between true and false memory. Our findings reveal that increased CT in the parietal lobe and middle occipital gyrus correlates with greater susceptibility to false memories, highlighting its role in integrating and manipulating memory information. Conversely, CT in the middle frontal gyrus and occipital pole was associated with enhanced accuracy in memory recall, emphasizing its importance in perceptual processing and encoding true memories. These results provide novel insights into the structural basis of memory errors and offer a foundation for future investigations into the neural underpinnings of memory reliability.

## 1. Introduction

Human memory is not a flawless recording device but a dynamic, reconstructive system optimized for efficiency [1]. Rather than storing exact replicas of experiences, our brains tend to extract and retain the essential meaning or “gist” of events [2]. This strategy allows for rapid encoding and retrieval, supporting adaptive functions like decision-making and learning in complex environments [1,3]. Critically, this process is not a design flaw but a feature of how our memory system evolved to prioritize relevance over precision. For example, the fuzzy-trace theory suggests that people form parallel memory traces—verbatim and gist—but rely more heavily on gist when making decisions or recalling past events, especially under time pressure or cognitive load [2].

However, this efficiency comes at a cost. By emphasizing generalized meanings over specific details, the memory system becomes susceptible to errors, such as false memories—recollections of events that either did not occur or occurred differently than remembered [3,4]. These errors are not simply failures but consequences of the same constructive mechanisms that typically serve us well. For instance, in the Deese–Roediger–McDermott (DRM) paradigm [4], participants often falsely recall or recognize semantically related words that were never presented, indicating the role of gist-based processing. Schema-driven encoding and retrieval further contribute to these distortions, as prior knowledge shapes what we attend to and remember, sometimes biasing or altering the original trace [5,6]. Recognizing that memory prioritizes efficiency over fidelity allows for a more nuanced appreciation of its adaptive strengths and vulnerabilities, with implications for fields such as eyewitness testimony, education, and clinical intervention [3,7].

The current study builds on previous functional magnetic resonance imaging (fMRI) research identifying functional neural correlates of true and false memory by examining whether gray matter structure in key regions of interest is also associated with memory accuracy. To our knowledge, this is the first study to investigate gray matter correlates of false memory.

Structural magnetic resonance imaging (sMRI) offers a non-invasive method for examining gray matter, which is an aggregate measure of neuronal cell bodies, dendrites, synapses, and glial cells [8,9]. Neurons with greater density of dendritic extensions, or arborizations, are highly specialized to receive sensory or motor information [10]. Advances in sMRI analysis techniques now allow for precise quantification of gray matter structure through metrics such as gray matter volume (GMV) and cortical thickness (CT). Research using CT has shown that structural brain networks often parallel functional organizational patterns, co-occur with myelination trajectories, and vary with experience and developmental stage [9,11,12]. Because sMRI is not task-dependent, it provides a more stable and broadly applicable measure of brain structure, which can complement and add to functional MRI (fMRI) findings [13].

### 1.1. Signal Detection Theory

Signal detection theory (SDT) was used to provide a highly sensitive measure of false memory task performance. SDT is a theoretical model that may explain how participants make decisions on the memory task when experiencing uncertainty [14,15,16]. For example, if a participant experiences a degree of uncertainty as to whether they had previously been shown a word and picture or just a word, one with a liberal response bias would answer that the stimulus has been seen before if it is even slightly familiar. Other participants may have a more conservative response bias and only indicate they had seen a picture of the item in cases of complete certainty.

For this examination, the *C* variable was used as a measure of response bias, wherein a low *C* value was an indication of liberal response bias and more frequent responses on the memory task. SDT informed the calculation of the *d*′ variable as a measure of the ability for an individual to correctly identify stimuli, despite the presence of distractor stimuli. Participants with a high value for *d*′ were less likely to respond to false alarms because they were better able to identify these as noise. SDT was used to identify two research hypotheses used during statistical analysis to understand the results of this examination. First, to identify if there was a correlational relationship between CT in ROIs and results on the false memory task. Second, to understand participants’ sensitivity to accurately detect signals in the presence of noise (*d*′).

### 1.2. Functional Correlates of False Memory

False memories arise from the reconstructive nature of memory, wherein the brain integrates sensory inputs, semantic associations, and prior knowledge. Neuroimaging studies have identified several brain regions involved in the formation and retrieval of false memories, often overlapping with those activated during true memory processes. Currently, there is little research that has examined the structural correlates of false memory. Marchewka et al. (2009) used voxel-based morphometry to examine whether gray matter volume (GMV) in various brain regions correlated with false memory during facial recognition tasks involving emotional content [17]. Zhu et al. (2016) examined whether GMV in the hippocampus and fusiform gyrus correlated with performance on a misinformation task where participants viewed various crime scenarios [18]. Their results suggested that GMV in these regions were involved in both true and false memories, though they differed in terms of whether they were associated with short-term versus long-term memories. We used these findings along with fMRI studies to inform upon regions of interest for the current study. Our study presents the first study examining cortical thickness correlates of false memory.

Kurkela & Dennis (2016) [19] conducted a meta-analysis of 28 event-related fMRI studies of false memory using Activation Likelihood Estimation (ALE) to identify consistent neural correlates across experiments. They found converging evidence that both true and false memories reliably activate the left inferior parietal lobule, medial prefrontal cortex, and bilateral hippocampus, supporting the idea that these regions underlie the general subjective experience of remembering, regardless of accuracy. However, true memories showed stronger activation in sensory-related areas (e.g., occipital cortex), whereas false memories were more likely to recruit frontal areas associated with monitoring and decision-making, such as the dorsolateral prefrontal cortex, which includes the middle frontal gyrus. This contrast highlights the differential involvement of sensory reactivation versus strategic retrieval processes in the generation of true versus false memories and supports the reconstructive nature of episodic memory.

The current study builds upon the data provided by two fMRI studies: Muncy and Kirwan (2020) [20] and Stephan-Otto et al. (2017) [21]. Stephan-Otto et al. (2017) [21] investigated brain activation during encoding and recall phases of memory formation. They hypothesized that visual processing regions—such as the lingual gyrus, middle occipital gyrus (MOG), and fusiform gyrus—would be active during encoding. Results showed activation in the inferior and superior parietal lobes, left middle frontal gyrus (MFG), and left inferior occipital gyrus (IOG) during false recalls. The lateral occipital cortex was associated with effective encoding of pictured stimuli. These findings support the hypothesis that the left MFG and parietal lobes contribute to distinguishing real from imagined images—functions linked to decision-making [22,23] and working memory [24,25,26].

Muncy & Kirwan (2020) [20] explored neural activity during memory retrieval using a lure discrimination task. In particular, they examined how mnemonic generalization—a cognitive process whereby memories for specific experiences are distorted through association with similar but incorrect information—impacts the integrity of original memory traces. Their goal was to identify brain regions activated during successful identification of target stimuli when presented alongside visually similar lures. They predicted activation in regions involved in visual attention and content processing, including the occipital cortex, hippocampus, angular gyrus, amygdala, and medial prefrontal cortex (mPFC) [26,27]. Results indicated that successful retrieval (Hits) activated the mPFC, angular gyrus, anterior middle temporal gyrus, and precuneus. Decreased activation during successful retrieval occurred in the left dorsal mPFC, insula, and inferior frontal gyrus—regions associated with self-referential processing and salience detection [20,22]. Retrieval of episodic memories also engaged the anterior insular cortex and right supramarginal gyrus [23,28].

Together, these studies suggest that the left MFG and parietal lobes support generalized recognition and discrimination processes in memory retrieval, while the occipital lobe is more directly associated with accurate recall of visual stimuli [20,21]. The findings from these studies have helped inform us about key regions of interest we included in our analyses, as listed below, which are supported by the current literature of functional neural correlates for false memory.

**Figure 1:**
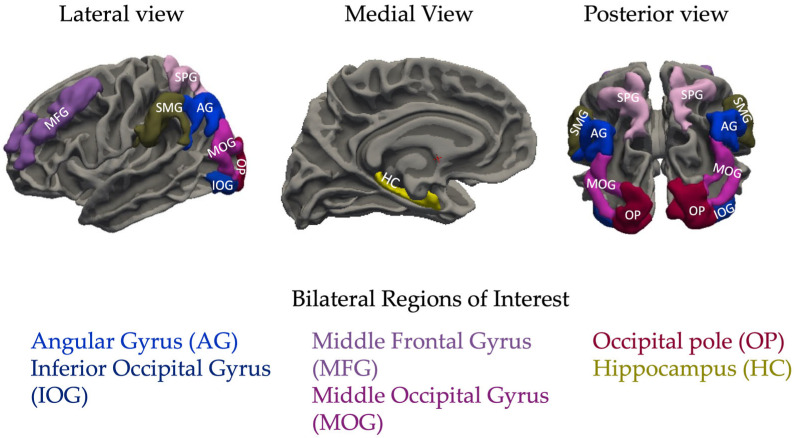
Neural regions of interest in the current study, based on fMRI findings of false memory. Lateral and medial views only show the left hemisphere, however our analyses included both hemispheres.

Occipital cortex ROIs included the bilateral middle occipital gyrus (MOG), inferior occipital gyrus (IOG), and the occipital pole (OP), which encompasses the primary visual cortex. Parietal cortex ROIs included the bilateral superior parietal lobule, angular gyrus (AG), and supramarginal gyrus (SMG). The MFG was included due to its established role in decision-making [21,22,23]. Recent primary literature identified cortical thickness (CT) as a valid and sensitive measure of gray matter structure, particularly because it often corresponds with other MRI, genotypic, and phenotypic measures and accounts for cortical folding, unlike total gray matter volume [29,30,31,32,33,34,35]. Figure 1 overviews bilateral regions of interest used in the current study based off fMRI studies.

#### 1.2.1. Frontal Lobe Regions

##### Middle Frontal Gyrus (MFG)

The middle frontal gyrus is associated with executive functions, including monitoring and decision-making during memory retrieval. Mixed findings are found in the literature in relation to false memory. A review conducted by Straube (2012) emphasized the role of lateral frontal regions including the MFG in the formation of true memories [36]. This is supported by Gutchess and Schacter (2012), who observed increased activation in the right middle frontal gyrus during false recognitions, implying enhanced monitoring efforts to resolve memory ambiguity [24]. This aligns with findings by Zhu et al. (2019) [26], who reported reduced prefrontal monitoring during conditions that elicited more false memories, highlighting the region’s role in memory accuracy. This is further supported by Marchewka et al. (2009) who found that false recognition was negatively correlated with GMV in prefrontal areas that included the MFG [17]. Additional studies have highlighted that connectivity between the right MFG and the left parahippocampal gyrus was associated with false recollection as compared to true recollection [37]. On the other hand, studies such as Bollinger et al. (2010) [38] have found that connections between the middle frontal gyrus and the fusiform gyrus were important for long-term memory success.

#### 1.2.2. Occipital Lobe Regions

Regions in the occipital cortex were selected as ROIs due to evidence that suggests these areas may be involved in the encoding of false memories. According to Stephan-Otto et al. (2017) [21], increased activation of visual cortices was noted when subjects were encoding words that were later falsely remembered. Also, the high visual imagery group showed increased activation in the occipital cortices compared to the low visual imagery group. Visual processing regions, such as the MOG and OP (primary visual cortex), are differentially engaged during true and false memories. Slotnick and Schacter (2004) [39] demonstrated that true memories elicited stronger activation in early visual areas compared to false memories, suggesting that sensory reactivation is more robust for accurate recollections.

1. Inferior Occipital Gyrus (IOG)

The IOG was selected as a region of interest due to evidence that suggests there is activation of the left IOG during encoding false memories [21]. These findings suggest that activation of regions in the occipital cortex may be involved in visual processing and are active during false memories of pictures. The synthesis of these findings led to the formation of the research hypothesis that increased CT in the MOG and IOG could lead to increased false responses on the memory task.

2. Middle Occipital Gyrus (MOG)

The MOG was selected as an ROI because evidence suggests that it plays a role in visual processing [21,27]. Gutchess and Schacter (2012) [24] further noted that activity in the middle occipital cortex increased with the number of related encoded items, indicating its role in processing visual similarities that may lead to false recognitions.

#### 1.2.3. Parietal Lobe Regions

The inferior parietal lobe, encompassing the AG and SMG, plays a pivotal role in memory retrieval and the integration of sensory information. For example, evidence suggests they are important in recognition processes [26,32,40] as well as episodic memory formation [40,41]. Gutchess and Schacter (2012) [24] found that both true and false recognitions activated the bilateral inferior parietal cortex, suggesting its involvement in reconstructive memory processes. Other studies have emphasized that these regions are more implicated in false recognition [36].

1. Angular Gyrus (AG)

The AG was selected as a region of interest because of its importance to semantic processing, comprehension, and memory retrieval, serving as a convergence zone for integrating multimodal information [42,43]. The functions of the AG are hypothesized to also include reasoning skills and language comprehension [44]. Another important consideration for use of this ROI is its implication in episodic memory recall, including in task-specific recollection [40]. These findings were synthesized to hypothesize that decreased CT in the SMG and AG may lead to decreased ability to distinguish real from imagined mental images and increase the incidence of responses to false alarms.

2. Supramarginal Gyrus (SMG)

The SMG contributes to phonological processing and working memory, playing a role in the manipulation and retrieval of verbal information [44]. The Stephen-Otto et al. (2017) [21] study identified activation in the right SMG and the superior parietal lobe during the presentation of words. The Muncy & Kirwan (2020) [20] study indicated this region is important for encoding episodic memories. Further evidence suggests that the strength of true memory was associated with the left SMG among other regions in the frontoparietal control network [20,26].

3. Superior Parietal Lobe (SPL)

Findings on the superior parietal lobe are mixed, with some findings suggesting it is important for both true and false memories. For example, Gutchess and Schacter (2012) [24] found that neural activity in posterior parietal regions which included the superior parietal lobe were more active for true minus false recognition. By contrast, Spets et al. (2021) [45] found that the right superior parietal lobule showed more activity during false memories versus misses. Dennis et al. (2012) [37] reported activation in the right superior parietal cortex during both true and phantom recollection, indicating its role in attentional and retrieval processes. Similarly, Okado and Stark (2003) [46] found similar activity for both true memories and false memories associated with activity in the left parietal cortex which included the superior parietal lobe. This activity was noted as possibly related to the perceived amount of retrieved information or how much contextual information was recovered or believed to be recovered.

#### 1.2.4. Temporal Lobe Regions

##### Hippocampus (HC)

The bilateral hippocampus was selected as a region of interest due to its well-established role in memory formation [47,48]. Functional neuroimaging studies have consistently demonstrated hippocampal engagement during the encoding and retrieval of both true and false memories [19,36]. These results are supported by structural neuroimaging findings: Zhu et al. (2016) found that GMV in the bilateral hippocampus was associated with both true and false short-term memories [18].

### 1.3. The Current Study

The goal of this study was to examine whether cortical thickness (CT) in specific brain regions is systematically associated with behavioral performance on a false memory task, with particular focus on memory sensitivity (*d*′) and response bias (*C*) as derived from signal detection theory (SDT) [16]. While prior research has largely emphasized functional differences between true and false memories [19,39], fewer studies have explored how gray matter structure contributes to individual differences in memory accuracy and distortion. This study aimed to address that gap by analyzing structural MRI data to identify regional CT correlates of false memory susceptibility.

Using publicly available data from Opener [49], we conducted a secondary analysis combining FreeSurfer-derived CT estimates [35] with behavioral performance on a false memory task. Greater CT in the left middle frontal gyrus and right occipital pole was associated with better discrimination, suggesting a role in strategic retrieval [50]. In contrast, increased CT in the supramarginal gyrus and middle occipital gyrus was linked to poorer discrimination, consistent with gist-based processing [51]. The resulting findings contribute to the scientific literature by advancing our understanding of the structural neural pathways involved in the formation of memory distortions.

## 2. Materials and Methods

### 2.1. Participants

Participant data collected from OpenNeuro [49] included 61 participants from both the Stephan-Otto et al. (2017) [21] https://openneuro.org/datasets/ds000203/versions/00001 accessed on 27 May 2024 and Muncy & Kirwan (2020) [20] https://openneuro.org/datasets/ds002242/versions/1.0.0 studies accessed on 6 September 2024. Specifically, the Stephan-Otto (2017) [21] study included 26 healthy adults (10 female; M = 37.3, SD = 9.1), and the Muncy & Kirwan (2020) [20] study included thirty-five participants (15 female, mean age = 23.1, SD = 2.2). All information about MRI acquisition and task details can be found in their publications and their OpenNeuro pages.

### 2.2. Behavioral Data

**Figure 2:**
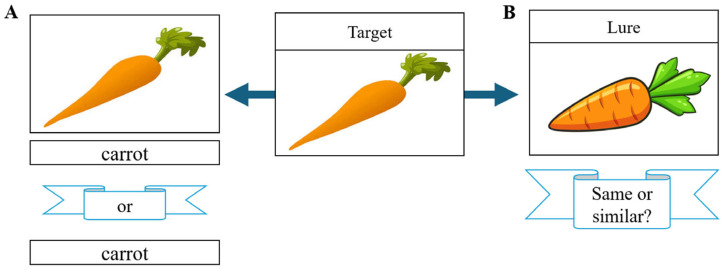
Overview of participant tasks. (**A**) Participants in the Stephan-Otto et al. (2017) [21] study were shown either a picture and its corresponding word or just the word. They were later presented with the word and asked if they had seen the image before. If the participant thought they saw an image when they had not, the trial was a false alarm. (**B**) Participants in the Muncy & Kirwan [20] study (2020) were shown an image in the study phase. During the test phase, they were either shown the same or a similar image. The trial was a false alarm if they identified a different image as the target image.

Participants in the Stephan-Otto et al. (2017) [21] study viewed 45 common item names either with or without a corresponding image during the encoding phase. During recall, they were asked whether they had previously seen a picture of each item. Trials in which participants falsely remembered seeing a picture were coded as false alarms. Similarly, Participants in the Muncy & Kirwan (2020) [20] study underwent three phases: a Study phase (categorizing 200 common objects as indoor/outdoor), Test 1 (discriminating between 100 targets and 100 lures), and Test 2 (choosing the correct image from target-lure pairs). Responses were coded as Hits, Correct Rejections (CRs), False Alarms (FAs), or Misses. See Figure 2 below for an example of these tasks.

To assess participants’ memory performance and their ability to discriminate between previously seen items and novel or similar lures, we applied principles of signal detection theory (SDT) [16]. SDT provides a model-based approach that separates sensitivity to stimulus differences (i.e., discriminability) from decision bias (i.e., response criterion), allowing us to more accurately evaluate memory performance beyond raw accuracy.

Participants’ responses were classified into hits (correctly identifying old stimuli), false alarms (incorrectly identifying new or lure stimuli as old), correct rejections, and misses. From these values, we calculated *d*′ (d-prime) as a measure of sensitivity and *C* as an index of response bias. *d*′ was computed as the standardized difference between the hit rate and the false alarm rate as follows:

*d*′ = *Z*(Hit Rate) − *Z*(False Alarm Rate) (1)

where *Z* represents the inverse of the cumulative distribution function of the standard normal distribution. A higher *d*′ reflects greater sensitivity or discrimination between old and new items. Response bias was calculated using the *C* criterion, given by the following:

*C* = −0.5 × [*Z*(Hit Rate) + *Z*(False Alarm Rate)] (2)

where more negative values indicate a liberal bias (tendency to respond “yes” or “same” to both targets and lures), and more positive values indicate a conservative bias (tendency to respond “no” or “similar” to both targets and lures) [52].

### 2.3. MRI Preprocessing

Structural data were preprocessed using FreeSurfer version 7 software, which is well-suited for accurately modeling data across different scanners and imaging protocols [35,53]. FreeSurfer’s measurements of cortical thickness (CT) and gray matter volume (GMV) have been cross-validated with histological analyses [54] and demonstrate high test–retest reliability [35]. The preprocessing pipeline included motion correction, averaging of T1-weighted images, skull-stripping, removal of non-brain tissue, intensity normalization, gray matter segmentation, white matter tessellation, automated topology correction, and cortical parcellation based on gyral and sulcal anatomy. FreeSurfer’s CT calculations are sensitive to submillimeter-level changes, while GMV measurements are limited by voxel resolution (1 × 1 × 1 mm in this study) [35]. All structural data underwent manual quality inspection. Data were preprocessed via the Open Science Pool provided by the Open Science Grid Consortium [55,56,57].

### 2.4. Data Analyses

A priori region-of-interest (ROI) analyses were conducted on bilateral regions including the middle occipital gyrus (MOG), inferior occipital gyrus (IOG), occipital pole (OP), hippocampus (HC), superior parietal lobe, angular gyrus (AG), supramarginal gyrus (SMG), and middle frontal gyrus (MFG). These regions were selected based off previous neuroimaging studies of false memory. We selected the Destrieux cortical parcellation atlas [58] since it includes more detailed regions of interest as compared to most other Freesurfer parcellation atlases. Statistical analyses were conducted in Jamovi Version 2.5 (2024) [59,60] to examine partial correlation and moderation analyses of these variables, which were all mean-centered. Estimated total intracranial volume (eTIV) was utilized as a standard control so that individual differences in brain size and volume would not contribute to significant findings. To correct for multiple comparisons, we applied false discovery rate (FDR) corrections [61].

## 3. Results

**Figure 3:**
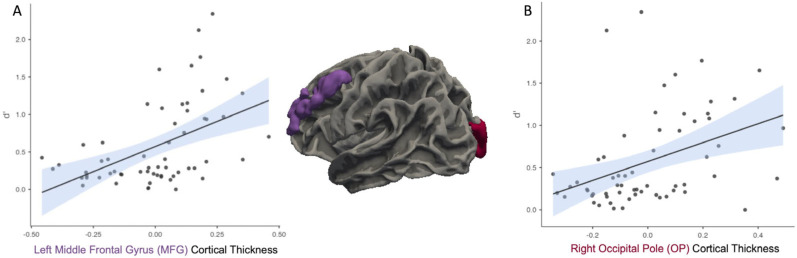
Greater cortical thickness in the left middle frontal gyrus (MFG; (**A**)) and right occipital pole (OP; (**B**)) is associated with higher memory sensitivity (*d*′). For both plots, each black dot represents an individual participant’s data point. The solid line shows the fitted regression line from the model. The shaded area around the line reflects the standard error of the estimate.

When controlling for eTIV, partial correlational analyses indicated that there was a significant positive relationship between *d*′ and CT in the left MFG, *r* = 0.49, *p* < 0.001 (Figure 3A) and CT in the right OP *r* = 0.39, *p* = 0.002 (Figure 3B). Both of these findings were still significant after FDR correction.

**Figure 4:**
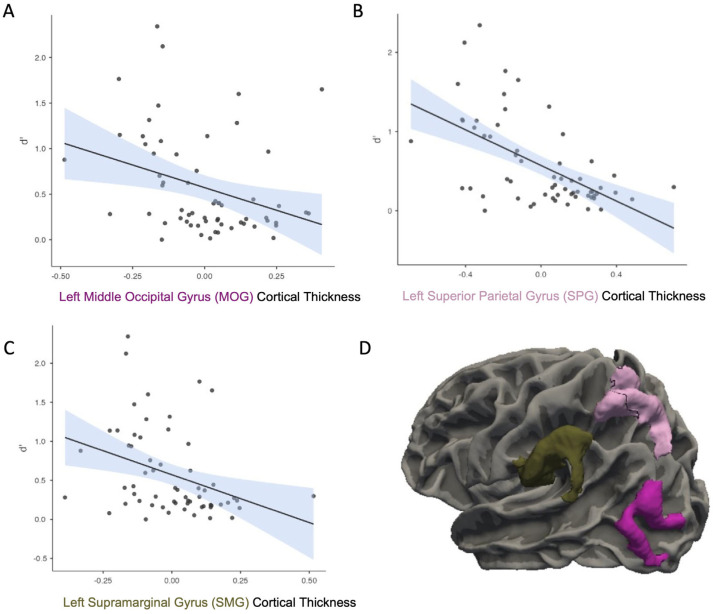
Cortical thickness in the left middle occipital gyrus (MOG; (**A**)), superior parietal gyrus (SPG; (**B**)), and supramarginal gyrus (SMG; (**C**)) was negatively correlated with memory sensitivity (*d*′). (**D**) Shows a visualization of the location of these brain regions. For all plots, each black dot represents an individual participant’s data point. The solid line shows the fitted regression line from the model. The shaded area around the line reflects the standard error of the estimate.

Other analyses indicated that the CT in the left MOG was negatively correlated with *d*′, *r* = −0.32, *p* = 0.013 (Figure 4A). CT in the left superior parietal lobe was negatively correlated with *d*′, *r* = −0.57, *p* < 0.001 (Figure 4B). CT in the left SMG was also negatively correlated with *d*′, *r* = −0.35, *p* = 0.007 (Figure 4C). All of these findings survived FDR correction. CT in the right AG was negatively correlated with *d*′, *r* = −0.29, *p* = 0.032. This right AG finding did not survive FDR correction.

**Figure 5:**
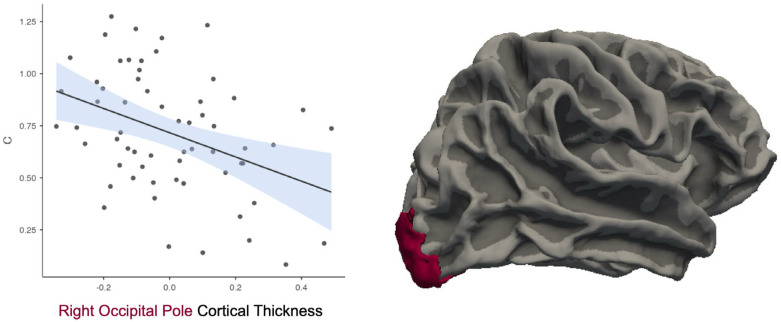
Greater cortical thickness in the right occipital pole (OP) is associated with a more liberal response bias (lower *C*). Each black dot represents an individual participant’s data point. The solid line shows the fitted regression line from the model. The shaded area around the line reflects the standard error of the estimate.

Analysis of the *C* variable indicated significant correlations with CT in the left AG, *r* = −0.29, *p* = 0.025. However, this finding did not survive FDR correction. The right occipital pole analyses indicated a significant negative correlation with *C*, *r* = −0.39, *p* = 0.002 (Figure 5), which survived FDR correction.

**Figure 6:**
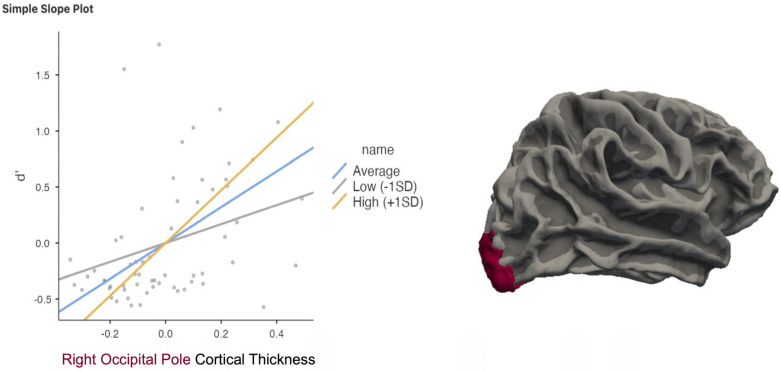
Moderation analysis indicated that individuals with a higher response bias (*C*) demonstrated a stronger positive correlation between memory sensitivity (*d*′) and cortical thickness (CT) in the right occipital pole (OP). Each black dot represents an individual participant’s data point. The solid line shows the fitted regression line from the model. The shaded area around the line reflects the standard error of the estimate.

Due to the disparate findings of the right occipital pole, we performed a post hoc moderation analysis to examine whether *C* moderated the relationship between *d*′ and CT. We found that *C* significantly moderated the relationship between CT in the right OP and *d*′ with *p* = 0.015 (Figure 6).

In sum, significant correlations that survived FDR correction between CT and *d*′ or *C* were found in six ROIs (Table 1).

## 4. Discussion

The current study represents the first sMRI investigation of cortical thickness correlates of false memory in these regions of interest. This study builds upon previous gray matter volume investigations into false memory in response to misinformation provided in crime scenarios [18] and emotionally charged false memories [17]. Drawing upon regions previously identified in sMRI and fMRI studies, this study aimed to determine whether differences in cortical thickness are associated with behavioral outcomes during a false memory task. Previous work has primarily focused on the functional underpinnings of false memory, with fMRI studies demonstrating that true and false memories engage many overlapping brain regions, including those related to memory, attention, and decision-making [19,20,21]. However, until now, the relationship between cortical thickness and false memory performance had not been explored.

Consistent with fMRI findings, the current sMRI analysis found that greater CT in the left middle frontal gyrus (MFG) was positively associated with *d*′, a signal detection theory (SDT) measure reflecting the ability to discriminate between targets and lures [36]. This supports prior interpretations of the MFG’s involvement in strategic retrieval monitoring and decision-making during memory tasks [21,22,23]. The positive association between MFG CT and *d*′ suggests that individuals with greater cortical thickness in this region are better equipped to distinguish true memories from false ones, likely due to more effective decision-related processing and discrimination of real versus imagined stimuli [24,25,36].

In contrast, negative correlations were observed between *d*′ and CT in several posterior regions, including the left middle occipital gyrus (MOG), left supramarginal gyrus (SMG), and left superior parietal cortex. These findings indicate that increased CT in these areas was associated with reduced ability to distinguish between targets and lures. This aligns with hypotheses derived from both Muncy & Kirwan (2020) and Stephan-Otto et al. (2017) [20,21], who found that parietal and secondary occipital cortices support visual processing and generalized recognition, as opposed to the recollection of detailed episodic content. These regions have consistently been implicated in false recollection [36] and gist-based retrieval, which increases susceptibility to false alarms [26,32,40].

The right occipital pole emerged as a region of particular complexity, showing divergent relationships with memory discrimination and response bias. We observed a positive correlation between CT and *d*′, indicating that greater gray matter microstructure in this region was associated with enhanced discrimination between targets and lures. At the same time, CT in the right occipital pole was negatively correlated with *C*, reflecting a more liberal response bias in individuals with thicker cortex in this area. To further examine this relationship, we conducted a moderation analysis to determine whether response bias (*C*) modulated the relationship between CT in the right occipital pole and *d*′. Results revealed a significant interaction: individuals with a more conservative response style (higher *C*) showed a stronger positive correlation between CT and *d*′, whereas those with a more liberal style (lower *C*) showed only a mild positive relationship. This suggests that the structural contribution of the right occipital pole to memory discrimination may depend on one’s decision strategy, with conservative responders deriving greater benefit from enhanced cortical thickness in early visual areas.

These findings align with and extend those of Slotnick and Schacter (2004) [39], who demonstrated that true memories elicit stronger activation in early visual cortices than false memories, consistent with more robust sensory reactivation during accurate recollection. The observed association between right occipital pole CT and *d*′, particularly among conservative responders, may reflect the anatomical substrate supporting this sensory-specific reactivation. This also supports the meta-analytic conclusions of Kurkela and Dennis (2016) [19], who found that early visual areas were more consistently recruited for true than false memories across fMRI studies. Our structural findings provide converging evidence that individual differences in cortical thickness in these regions are not only behaviorally relevant but also interact with response style to shape memory accuracy. This finding underscores the importance of accounting for response style when interpreting structure–function relationships, as the effectiveness of certain brain regions in supporting memory performance may depend on individual decision strategies. It also reinforces the value of incorporating SDT metrics in neuroimaging analyses to disentangle sensitivity from bias—two distinct but interacting components of cognitive performance.

Further, we found a negative correlation between CT in the left AG and the SDT measure *C*, which reflects response bias. Specifically, participants with greater CT in this region showed a more liberal response style (lower *C* values), indicating a tendency to respond affirmatively in ambiguous situations, however this finding did not survive FDR correction. This liberal bias may partially account for the increased false alarms in individuals with thicker cortex in these areas, suggesting that structural features of these regions not only relate to perceptual and mnemonic processes but also to decision thresholds and task response styles.

These findings conceptually replicate and expand upon earlier fMRI work indicating that false memories were associated with increased activation in the occipital cortex and inferior and superior parietal lobes, including the AG and SMG [20,21]. These regions were proposed to support visual imagery and mnemonic generalization, respectively. Our structural findings complement these interpretations: increased CT in regions were usually tied to visual perception and generalization was associated with poorer discrimination (lower *d*′) and greater liberal response bias (lower *C*), possibly due to stronger encoding of visual gist information or more inclusive response criteria.

Importantly, the present study incorporates SDT metrics to distinguish between memory sensitivity (*d*′) and response tendencies (*C*), allowing for more nuanced interpretations of behavioral outcomes. While past literature has focused primarily on the number of false alarms as evidence of memory failure, our findings suggest that these may also reflect individual differences in task approach. For instance, a participant with a more liberal response bias may appear to experience more false memories, not necessarily due to impaired memory, but due to a general inclination to respond “yes” when uncertain. This interpretation has implications for studies of cognitive aging, expertise, and other individual differences. Prior MRI experience or familiarity with similar tasks may also influence *d*′, potentially inflating performance in some participants. Future studies should account for such behavioral covariates.

The current study also builds upon the fMRI meta-analytic work of Kurkela & Dennis (2016) [19], who identified consistent activation of the MFG, parietal regions, and medial prefrontal cortex during both true and false memories. Our structural results correspond well with these functional findings. Notably, while MFG CT was associated with better memory discrimination, CT in parietal and occipital regions was related to increased false alarms and liberal bias, echoing the functional dissociation between sensory-specific and strategic retrieval regions highlighted in the fMRI literature.

Moreover, the methodology employed here extends prior fMRI-based investigations by leveraging the Freesurfer processing pipeline to assess cortical thickness from sMRI data archived on OpenNeuro. This represents a novel application of publicly available structural data and provides a cost-effective, replicable framework for linking brain morphology with cognitive performance. The successful use of sMRI data to mirror fMRI-derived regions of interest suggests that CT may serve as a structural proxy for functional activation in studies of episodic memory.

While prior work such as Soch et al. (2022) [62] used GMV rather than CT to examine sMRI data, their focus was on age prediction rather than memory discrimination. Similarly, Zhu et al. (2016) and Marchewka et al. (2009) examined GMV in participants who were exposed to highly emotional content such as crime scenarios [18] and emotional faces [17]. Thus, the present study is the first to link CT in specific regions to performance on a false memory task using SDT metrics. This is an important distinction since CT has been shown to be a more sensitive measure as compared to GMV, as it allows for sub-millimeter resolution and accounts for cortical folding [33,35]. This approach not only enhances the interpretability of behavioral data but also lays a foundation for future studies to explore how individual variability in CT may predict memory performance in broader cognitive domains, such as reasoning, attention, or language acquisition.

Despite these contributions, the present study is not without limitations. As the first of its kind, these findings require replication and expansion with larger samples and additional memory paradigms. Further research could integrate multiple imaging modalities (e.g., diffusion tensor imaging, resting-state fMRI) to examine how structural features interact with connectivity and activation patterns during memory tasks. Additionally, incorporating demographic and experiential factors (e.g., education level, task familiarity) may help disentangle anatomical from behavioral sources of variability.

## 5. Conclusions

In summary, this study provides strong preliminary evidence that CT in frontal, parietal, and occipital cortices is systematically related to behavioral outcomes on a false memory task. Greater CT in the left MFG and the right OP containing the primary visual cortex were associated with better memory discrimination, while increased CT in posterior regions—including the SMG, SPG, and MOG—was linked to poorer discrimination and more liberal response bias. These results suggest a structural dissociation between regions supporting strategic retrieval and those involved in gist-based processing. Notably, the right occipital pole exhibited a more complex pattern: CT in this region was positively related to memory sensitivity but negatively related to response bias, and further analysis revealed that the relationship between CT and sensitivity was moderated by bias. These results may reflect the role of sensory reactivation in supporting accurate recollection, as previously observed in functional studies [39], and are broadly consistent with meta-analytic evidence that early visual regions are recruited more reliably during true than false memories [19]. Together, these findings bridge functional and structural neuroscience studies and highlight the utility of SDT measures in dissecting the contributions of memory sensitivity and decision bias to false memory formation. The novel integration of sMRI, Freesurfer, and SDT analysis opens new avenues for investigating how brain structure underlies individual differences in memory and decision-making, with potential applications in education, aging, and clinical neuroscience.

## Figures and Tables

**Table 1:** Results of statistical analyses.

Brain Region of Interest	*d*′ *r*-Value	*d*′ *p*-Value	*C r-*Value	*C p*-Value
Left AG		n.s. ^a^	−0.29	*p* = 0.025 ^a,^*
Left HC		n.s. ^a^		
Left MFG	0.49	*p* < 0.001 ^a,^*^,^†		n.s.
Left MOG	−0.32	*p* = 0.013 ^a,^*^,^†		n.s.
Left OP		n.s. ^a^		n.s.
Left SMG	−0.35	*p* = 0.007 ^a,^*^,^†		n.s.
Left SPG	−0.57	*p* < 0.001 ^a,^*^,^†		n.s.
Right AG	−0.28	*p* = 0.032 ^a,^*		n.s.
Right HC		n.s. ^a^		n.s.
Right MFG		n.s. ^a^		n.s.
Right MOG		n.s. ^a^		n.s.
Right OP	0.4	*p* = 0.002 ^a,^*^,^†	−0.39	*p* = 0.0024 ^a,^*^,^† n.s.
Right OP X *C* Moderation		*p* = 0.015 ^b,^*^,^†		
Right SMG		n.s. ^a^		n.s. ^a^
Right SPG		n.s. ^a^		n.s. ^a^

Note. ^a^ Statistical analysis: partial correlation with eTIV as a control variable. ^b^ Statistical analysis: moderation analysis. * *p*-values < 0.05 were considered significant. † significant after FDR corrections.

## Data Availability

The original data presented in the study are openly available on OpenNeuro: https://openneuro.org/datasets/ds000203/versions/00001 (accessed on 27 May 2024) and https://openneuro.org/datasets/ds002242/versions/1.0.0 (accessed on 6 September 2024).

## Abbreviations

The following abbreviations are used in this manuscript:

AG	Angular Gyrus
ALE	Activation Likelihood Estimation
*C*	Conservative Response Bias
CR	Correct Rejection
CT	Cortical Thickness
*d*′	Response Sensitivity
DRM	Deese–Roediger–McDermott
eTIV	Estimated Total Intracranial Volume
FA	False Alarm
FDR	False Discovery Rate
fMRI	Functional Magnetic Resonance Imaging
GMV	Gray Matter Volume
IOG	Inferior Occipital Gyrus
MFG	Middle Frontal Gyrus
MOG	Middle Occipital Gyrus
mPFC	medial Prefrontal Cortex
MRI	Magnetic Resonance Imaging
OP	Occipital Pole
PFC	Prefrontal Cortex
ROI	Region(s) of Interest
SDT	Signal Detection Theory
SMG	Supramarginal Gyrus
sMRI	Structural Magnetic Resonance Imaging
SPG	Superior Parietal Gyrus (also Superior Parietal Lobe)
